# The change in rumen temperature post-drink can be used to estimate water intake in sheep

**DOI:** 10.1093/jas/skaf368

**Published:** 2025-10-23

**Authors:** Georgia E Welsh, Serina N Hancock, Luoyang Ding, Dominique Blache, Shane K Maloney

**Affiliations:** College of Environmental and Life Sciences, School of Agriculture, Murdoch University, Murdoch, Western Australia, 6150, Australia; College of Environmental and Life Sciences, School of Agriculture, Murdoch University, Murdoch, Western Australia, 6150, Australia; School of Human Sciences, Anatomy and Physiology, The University of Western Australia, Crawley, Western Australia, 6009, Australia; School of Human Sciences, Anatomy and Physiology, The University of Western Australia, Crawley, Western Australia, 6009, Australia; School of Human Sciences, Anatomy and Physiology, The University of Western Australia, Crawley, Western Australia, 6009, Australia

**Keywords:** rumen temperature, sheep, water intake, thermodynamics

## Abstract

In ruminants, water ingestion causes an immediate and measurable decline in rumen temperature. While this physiological response is well established, its application for estimating water intake has not previously been investigated. This study assessed the suitability of a thermodynamic modelling approach to estimate water intake in sheep using intra-ruminal temperature data. A fluid calorimetry equation was first validated under controlled laboratory conditions, then applied to data from six sheep fitted with intra-ruminal temperature loggers. Animals were offered water of known volume and temperature, and intake estimations were calculated using the temperature drop, baseline rumen temperature, water temperature, and estimated rumen volume based on liveweight. To improve volume estimation, additional rumen volume and liveweight data from a separate group were used to generate a generalised prediction equation for rumen volume. The approach underestimated the volume of water consumed, and so we developed a correction factor to account for physiological variation in the effective rumen volume at the time of drinking. Using the effective rumen volume in calculations resulted in estimated intake volumes that generally aligned with the measured values (*P < *0.01). Our results demonstrate that measured changes in intra-ruminal temperature can be used to estimate water intake, offering a promising tool for precision livestock monitoring in extensive grazing systems.

## Teaser

Rumen temperature loggers offer a promising way to estimate the volume of water intake in sheep, supporting precision livestock monitoring in extensive grazing systems.

## Graphical abstract

**Graphical Abstract:**
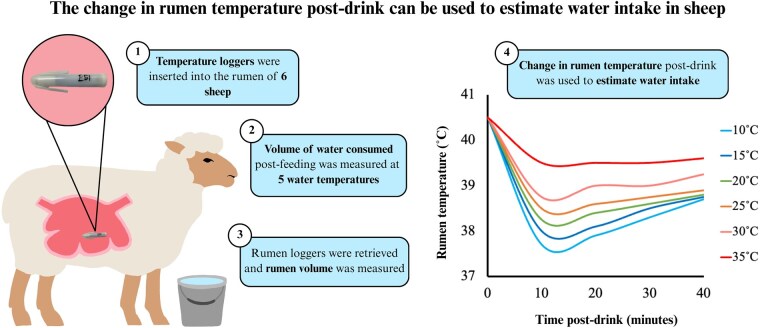

## Introduction

Thermal stress occurs when a homeothermic animal must increase evaporative heat loss (during heat stress) or metabolic heat production (during cold stress) to maintain heat balance (IUPS Thermal Commission, 2003). In sheep, reliance on evaporative cooling increases when ambient temperature exceeds 30 °C, and evaporation becomes the primary means to dissipate metabolic and environmental heat as air temperature approaches body temperature (Blaxter et al., 1959; Hopkins et al., 1978; Kumar et al., 2017). If evaporative losses are not replaced by water consumption, dehydration occurs (Wilson, 1970). Hence, sheep that are exposed to heat stress will drink more water (Barnes et al., 2004; Tüfekci and Sejian, 2023). A method to monitor water intake in the field could provide valuable insight into the physiological responses of sheep to heat stress and support strategies to reduce dehydration and associated production losses. The methods that are used to measure water intake in livestock are typically restricted to specialist research or feedlot settings, where animals are electronically identified, and water consumption is recorded using flow meters or weight-based systems (Mesgaran et al., 2020; Tang et al., 2021). While accurate, these systems are costly, require infrastructure and technical support, and are not practical for extensive grazing systems.

When a ruminant consumes water, the fluid will generally be cooler than the contents in the rumen, which leads to an immediate decline in rumen temperature (Cooper-Prado et al., 2011; Vesterdorf et al., 2022). Although some water bypasses the rumen and enters the abomasum (Woodford et al., 1984b; Cafe and Poppi, 1994), most enters the rumen, and temperature typically does not return to baseline for 60 to 90 minutes. Recovery depends on factors such as the volume and temperature of the water consumed, the rate of drinking, rumen fill and fermentation activity, motility, fluid passage, body size, hydration status, and ambient temperature (Brod et al., 1982; Woodford et al., 1984b; Cafe and Poppi, 1994; Bewley et al., 2008). Brod et al. (1982) reported that colder water produced a greater decline in rumen temperature, while Cantor et al. (2018) showed that in cattle, the magnitude of the decline also depended on the volume ingested. These findings suggest that, if water temperature is known, the change in rumen temperature could be used to estimate water intake.

Thermodynamic principles underpin the temperature changes that occur in the rumen after water intake. Richmann’s law of mixtures dictates that when liquids of different temperatures are combined, the final equilibrium temperature is determined by the weighted average of their mass, the specific heat capacity of the mixtures, and the initial temperatures (Richards, 2007; Cengel et al., 2020). In that context, the rumen and the ingested water could be treated as a thermal system in which heat is exchanged until equilibrium is reached, with no net loss or gain of energy beyond the system boundary. When a sheep consumes water, the ingested fluid mixes with rumen contents, and the change in temperature of the mixture can be predicted from these principles. Following ingestion, the return of rumen temperature toward baseline is governed by Newton’s law of cooling, which states that the rate of heat exchange between an object and its environment is proportional to the difference in temperature between them (Cengel et al., 2020). In this case, the cooling effect of water diminishes as the rumen contents equilibrate with surrounding tissues. Together, these principles provide the theoretical foundation to estimate water intake from the profile of rumen temperature.

In this study, intra-ruminal temperature loggers provide high-resolution measurements of the temperature change that is caused by a drinking bout (Cantor et al., 2018; Vesterdorf et al., 2022). The objective of this study was to test whether data on rumen temperature can be used to estimate water intake in sheep using a thermodynamic modelling approach. We hypothesised that the volume of water consumed could be estimated from the magnitude of temperature decline, provided that the temperature of the ingested water and rumen volume were known. By developing and refining this approach, we aim to develop an algorithm to estimate the volume of water that is consumed by sheep from the change in rumen temperature caused by the drink. The algorithm is intended for application in real-world conditions, offering a practical tool to monitor water intake in sheep under heat stress. Enabling the early detection of insufficient drinking and abnormal patterns ultimately supports strategies to minimise dehydration, reduce the risk of heat-related mortality, and improve welfare and productivity in the Australian sheep industry.

## Materials and Method

The research was approved by the Murdoch University Animal Ethics Committee (Permit number: R3403/22). All procedures were performed in accordance with the guidelines of the Australian Code of Practice for the Use of Animals for Scientific Purposes (National Health and Medical Research Council, 2021).

### Testing a model of fluid calorimetry

The mixing of two fluids at different temperatures is described by Richmann’s law of mixtures, which states that the final equilibrium temperature is the weighted average of the components according to their mass, specific heat capacity, and initial temperature (Cengel et al., 2020). Assuming that the density of the consumed water and the contents that are already in the rumen are approximately 1 kg·L^−1^ and that the specific heat capacity of each is close to that of water, the relationship simplifies to:


(Equation 1) $$T_{1+2}=\frac{\left(V_{1}\times T_{1}\right)+\left(V_{2}\times T_{2}\right)}{V_{1}+V_{2}}$$

**Figure 1.**
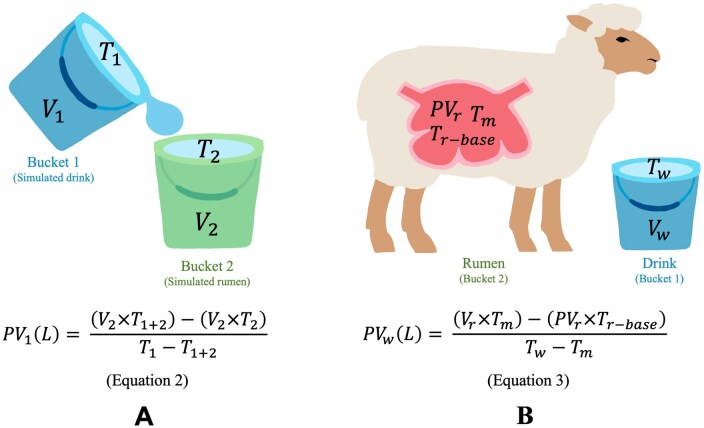
(A) Application of the thermodynamic equation in a controlled experiment to simulate the dynamics of water intake in sheep, where *T_1_* and *T_2_* are the temperature of Bucket 1 (simulated drink) and Bucket 2 (simulated rumen), respectively, *V_1_* and *V_2_* are the volume of the simulated drink (Bucket 1) and the simulated rumen (Bucket 2), *T_1 + 2_* is the temperature of the mixture, and *PV_1_* is the predicted volume of the drink. The equation in Fig. 1A (referred to as Eq. 2) is a rearrangement of the terms in Eq. 1 to solve for the volume of Bucket 1 (predicted volume of the “drink”). (B) Application of the thermodynamic equation to solve for water intake in sheep (Eq. 3), where *T_w_* is the temperature of the water, *T_r-base_* is the baseline temperature of the rumen, *T_m_* is the minimum temperature of the rumen after a drinking bout (equivalent to *T_1 + 2_* in Fig. 1A), *V_r_* is the volume in the rumen, and *PV_w_* is the predicted volume of water consumed.

An initial laboratory experiment with buckets of water was conducted at room temperature (24 °C), prior to an animal house experiment, to assess the applicability of a thermodynamic equation. Equation 1 was used to calculate the temperature of the mixture (***T_1 + 2_***), using the volume (***V****)*, and the temperature (***T***), of each fluid. The following experimental design mimicked the methodology that was later used in the animal house, as shown in Fig. 1. A bucket of water (10 L Metal Bucket, Pinnacle Hardware, Cranbourne West, Vic, Australia) was prepared with volumes varying between 4.25 and 5.9 L (***V_2_***) of water at close to 40 °C (***T_2_***), and another bucket of water with volumes varying between 1.2 and 2.3 L (***V_1_***) at different temperatures (***T_1_*** = 10 °C, 15 °C, 20 °C, 25 °C, and 30 °C). The *V_1_* mimicked the drinking episode, and *V_2_* mimicked the rumen. The water temperature was measured using a Radiospares PRO Handheld Digital Thermocouple (calibrated against a certified Omega Centre 376 Precision Resistance Temperature Detector). Each bucket was weighed (AND UC-321 Scale). Immediately after weighing, *V_1_* was poured into *V_2_* to create a mixture (***V_1 + 2_***). The *T_1 + 2_* was recorded once it had reached a steady value, and the volume of the mixture was weighed. The procedure was repeated six times for each *T_1_*.

We predicted the drink volume by rearranging the thermodynamic equation to solve for the volume of bucket 1 (which we call predicted volume 1; ***PV_1_***: Eq. 2 in Fig. 1A). To assess the applicability of Eq. 2, *PV_1_* was compared to the measured *V_1_* by regression analysis. Using the same principles, the volume of the water that was consumed by a sheep was calculated, as shown in Eq. 3 in Fig. 1B.

### Animal house experiment

The experiment was conducted at the animal house facility at Murdoch University in Perth, Western Australia, during late winter. Ambient temperature and relative humidity were measured every five minutes using two HOBO^Pro V2^ Temperature and Humidity Data Loggers (U23-0001), positioned 2.5 metres above ground level in the centre of the animal house facility. Water vapour pressure was calculated using the temperature and humidity values from each logger using the standard saturation vapour pressure equation and relative humidity adjustment. It was not included as a predictor in the models. For each experimental session (n = 34), the average ambient temperature, relative humidity, and calculated water vapour pressure were obtained by averaging all five-minute recordings across the session from each logger. The final value used for analysis was the mean of the two loggers. Three Merino and three Suffolk-Merino wethers (Crossbred), ranging in body mass from 43 to 59 kg with an average of 49 kg, aged 1–2 years, were housed in individual pens that were 2 m long by 1 m wide. Over a 12-day period on different days, each sheep was offered a known volume of water at one of six test temperatures: 10 °C, 15 °C, 20 °C, 25 °C, 30 °C, or 35 °C in the morning or the afternoon. The temperature of the water offered was randomised using a Latin square design, resulting in each sheep being offered each test temperature once in the morning and once in the afternoon. Water was provided at 0800 h and removed at 1000 h for morning sessions, and again provided at 1500 h and removed at 1700 h for afternoon sessions. Water was withheld overnight when testing in the morning trial, and between morning and afternoon when testing in the afternoon. A maintenance diet of Milne Feed EasyOne pellets (11.0 MJ ME/kg DM, 14.5% crude protein, 20% crude fibre) was provided twice a day, one hour before the commencement of each session.

### Water consumption and temperature measurement

The temperature of the rumen in each sheep was measured using a temperature logger (model ½AAA, Bryn O Morgan Industries, Perth, Australia) encased in an empty Rumensin capsule (Argenta P/L, New Zealand). The capsule was administered using an oral applicator one day before the experiment commenced. The loggers were calibrated against a certified Center 376 platinum resistance thermometer at four temperatures that covered the range of expected measurements before encapsulation. The loggers measured rumen temperature every 15 seconds throughout the experiment. This high temporal resolution was selected to capture rapid changes in rumen temperature that are associated with drinking events and to support the development of an algorithm intended for real-world field application.

A Radiospares PRO Handheld Digital Thermocouple (calibrated against a certified Center 376 Precision Resistance Temperature Detector) was used to obtain the water temperature in each bucket (Pinnacle Hardware 10 L Metal Bucket) immediately before and after each drinking session. Water temperature was measured using two temperature loggers in each bucket that recorded the temperature every 15 seconds. Those data were later used to obtain the average water temperature in each bucket for each session. Each bucket was weighed (FG Series Trade-Approved Platform Scale, 30 kg × 0.01 kg, Australian Science, Sydney, Australia) and offered for a 10-minute drinking session. After 10 minutes, the bucket was removed and weighed to determine the volume of water consumed.

The intraruminal temperature loggers were retrieved at slaughter after the experiment, recalibrated at four temperatures, and downloaded using LoggerMate V3.0 (Bryn O Morgan Industries, Perth, Australia). The average reading from each logger during each calibration step, and the calibrated temperature that was read from the certified thermometer, was used to create a unique calibration equation for each logger. That equation was then applied to the raw data before analysis. The time of the first drink was obtained by direct observation during the experiment and validated using a general-fixed threshold of 37.7 °C on the temperature trace to indicate a drink (Cantor et al., 2018). For each drinking event, a reference time point was defined based on two criteria: (a) the time at which drinking was visually observed and (b) the point at which the rumen temperature began to decline rapidly, identified using a threshold of at least 0.5 °C decrease within 15 seconds. The two minutes of data preceding this reference point were used to calculate the baseline rumen temperature (***T_r-base_***). Data from the subsequent 25 minutes were then used to determine the minimum rumen temperature (***T_m_***). Recovery time was defined as the interval from the reference point to the time when rumen temperature returned to *T_r-base_*. The recovery of rumen temperature after a drinking event was interpreted in the context of Newton’s law of cooling, which states that the rate of heat exchange between a body and its environment is proportional to the difference in temperature between them. In this application, the cooling effect of ingested water diminishes as the rumen contents equilibrate with the surrounding body tissues, providing the theoretical basis for the gradual return of rumen temperature toward baseline (Richards, 2007; Cengel et al., 2020). The temperature of the consumed water (***T_w_***) was calculated as the average temperature of the water in the bucket recorded by two loggers at the time of the first observed drink.

### Drinking behaviour

The individual drinking behaviour was monitored using three CCTV cameras (Efocus CCTV Security System 1080P) mounted 2.5 metres above each pen. The drinking behaviour was also monitored in real-time by human observers.

### The prediction of water consumption from the change in rumen temperature

The predicted volume (***PV_w_***) of each drink for each drinking bout was estimated using Eq. 3 (Fig. 1B), with the following inputs:

- Physiological rumen volume of each sheep (***V_r_***)
- Baseline rumen temperature from the temperature logger in the rumen before a drink (***T_r-base_***)
- Temperature of the water consumed, as measured by the loggers in the bucket (***T_w_***)
- Minimum rumen temperature from the temperature logger in the rumen soon after the drinking bout (***T_m_***)

### Rumen dissection and rumen volume

At the completion of the experiment, the sheep were fasted for 24 hours before being sent to an abattoir, where they were slaughtered, and their rumens were collected. The rumens were transported on ice to a laboratory for dissection. Each rumen was emptied, the logger was removed, and the contents were weighed (WEV 300 kg × 100 g Shipping Scale). The volume of the rumen contents (termed the physiological volume) was measured using a measuring cylinder. To expand the dataset on rumen volume, the rumens from an additional 20 sheep were collected after those sheep had been weighed and slaughtered, and their rumen volume was measured. These animals were adult Merino ewes, aged approximately 5 years, with a mean body mass of 63.6 ± 6.7 kg. With the data from all 26 sheep, a relationship between body mass (***BM***) in kilograms and physiological rumen volume was established using linear regression to predict the physiological rumen volume (***PV_r_***).

### Statistical analysis

All statistical analyses were conducted using R Studio (Integrated Development for R, RStudio, PBC, Boston, Massachusetts, United States) and Python (Python Software Foundation, Wilmington, Delaware, United States). Linear and quadratic regression were used to assess: (i) the bucket experiment (the predicted vs. measured volume of Bucket 1), (ii) the relationship between body mass and *PV_r_*, (iii) the predicted vs. measured volume of a drink, and (iv) the relationship between *PV_r_* and an effective rumen volume (*EV_r_*; see results). Quadratic (nonlinear) terms were evaluated for (ii) and (iii), however, they did not improve the fit, and they were therefore excluded from the final models. Pearson’s correlation quantified bivariate associations. An ANOVA tested the effects of water temperature, breed, and body mass on water consumption, with time of day and ambient temperature as additional factors. Pen and day were included as blocking terms. Predictive performance was summarised using the coefficient of determination (*r*^2^), adjusted *r*^2^ (Adj. *r*^2^), Mean Squared Error, Root Mean Squared Error, and Mean Absolute Error. The correction factor applied to the *EV_r_* equation was tested for its effect on prediction accuracy. Statistical significance was set at *P *< 0.05 for all analyses.

## Results and Discussion

### Testing the thermodynamic model of fluid calorimetry

**Figure 2.**
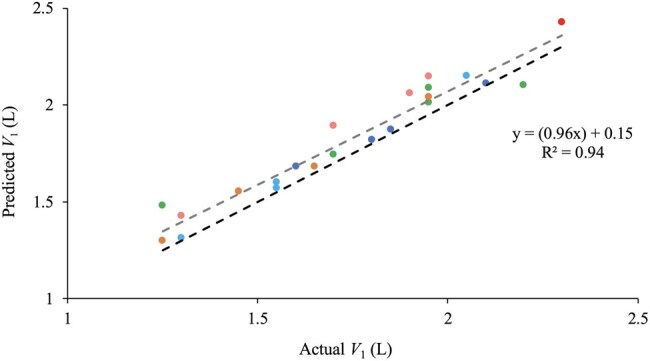
The measured volume of Bucket 1 (***V_1_***) and the predicted volume of Bucket 1 (***PV_1_***) that was calculated from the measured change in temperature of the mixture, using the thermodynamic equation (Fig. 1A; Eq. 2) across the five temperature treatments, 10 °C (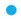), 15 °C (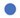), 20 °C (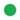), 25 °C (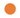), and 30 °C (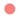), against a line of identity (y = x;---) and linear line of regression (---).

Equation 2 reliably predicted the volume of Bucket 1 when two buckets of water were mixed (*V_2_*; *r*^2^ = 0.94, *P *< 0.01, y = (0.96x) + 0.15; Fig. 2). On average, Eq. 2 overestimated the volume of Bucket 1 by 0.08 L compared to the measured volume in that bucket. Figure 2 supports the validity of the thermodynamic model, which was later used to predict the volume of water consumed by sheep based on changes in rumen temperature under the assumption that similar thermodynamic principles would apply in a biological context.

The temperature of the mixture in Bucket 2 ranged from 33 °C to 39 °C across all five temperature treatments, which was considerably warmer than the ambient temperature of the laboratory (24 °C). This temperature difference resulted in continuous heat loss from the buckets to the surroundings, further facilitated by the metal composition of the bucket, which has a high thermal conductivity. An assumption of the thermodynamic model is that no heat is lost or gained by the mixture, which is not the case in the bucket experiment (Mitrovic, 2022). Therefore, it is not surprising that the equation slightly overestimated the volume of the “drink” (Bucket 1) because the heat that was lost from the mixture was effectively added to the volume of the simulated drink. In contrast, during the animal house experiment, after a drink, the rumen will gain heat from the body of the sheep. This fundamental difference between heat loss from the metal container and heat gain by the rumen may introduce errors in the application of Eq. 3.

### The prediction of physiological rumen volume

**Figure 3.**
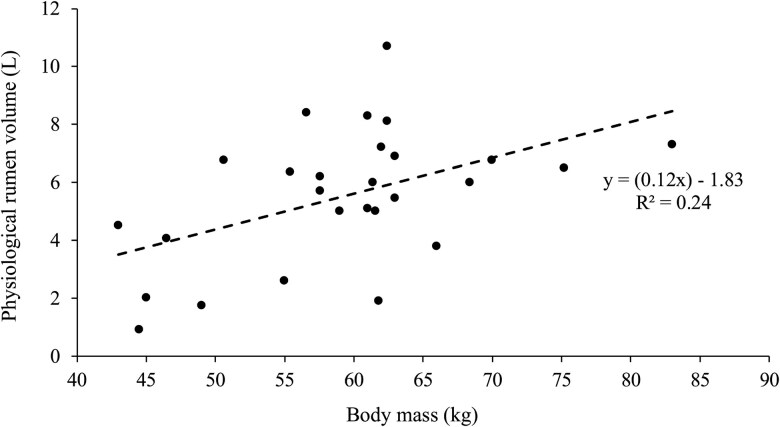
The relationship between body mass (kg) and the measured physiological rumen volume (L) in twenty-six sheep, as measured post-slaughter.

A Pearson correlation test revealed that BM and *V_r_* were significantly related (*r *= 0.49, *P *< 0.01), albeit with some variation. A linear regression model explained 24% of the variance in *V_r_* (*r*^2^ = 0.24, Adj. *r*^2^ = 0.21) with a Mean Squared Error of 3.9, a Root Mean Squared Error of 1.98 L, and a Mean Absolute Error of 1.57 L (Fig. 3):


(Equation 4) $$PV_{r}\;(L)=(0.12\times BM)-1.83$$

A quadratic regression was also tested, and provided only marginal improvement (*r*^2^ = 0.30, Adj. *r*^2^ = 0.24, Root Mean Squared Error = 1.90 L, Mean Absolute Error = 1.50 L), indicating limited gain in explanatory power relative to the increase in model complexity. Given the marginal improvement, the linear regression model was considered sufficient and retained for interpretability. Due to its practicality and biological relevance, the body mass-based Eq. 4 was used to calculate the predicted physiological rumen volume for each sheep (***PV_r_***) in the animal house experiment rather than the *V_r_* for each individual, as the differences were minor. Body mass is a readily accessible metric that could provide useful insights into rumen volume, even though it does not account for all sources of variation (Goopy et al., 2014).

### The prediction of the volume of consumed water from the change in rumen temperature

**Figure 4.**
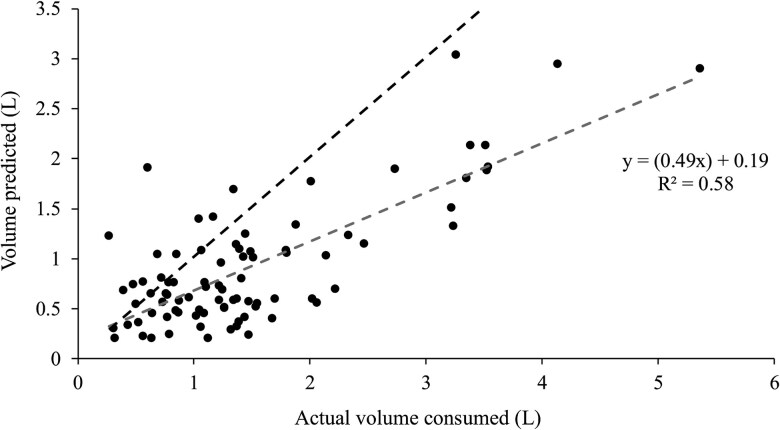
The volume of water consumed (L) during an individual drinking bout and the predicted volume of water consumed (L), calculated using the physiological rumen volume (***PV_r_***; Eq. 4) in the thermodynamic equation (Fig. 1B; Eq. 3), with the line of identity (y = x;---) and the linear regression line (**---**) for comparison.

Regression analysis of the predicted volume of water consumed, using the *PV_r,_* against the actual volume of water consumed, yielded a moderate fit for the data (*r*^2^ = 0.58, Adj. *r*^2^ = 0.57, Mean Squared Error = 0.39, Root Mean Squared Error = 0.63 L, Mean Absolute Error = 0.50 L; Fig. 4). On average, the linear model explained 58% of the variance, although Eq. 3 (Fig 1B) consistently underestimated the volume of the drink by approximately half. A quadratic regression was also tested and provided only marginal improvement (*r*^2^ = 0.60, Adj. *r*^2^ = 0.59, Mean Squared Error = 0.37, Root Mean Squared Error = 0.61 L, Mean Absolute Error = 0.48 L), indicating limited gain in explanatory power relative to the increase in model complexity. Given the quantifiable nature of water temperature, water volume, minimum rumen temperature, and baseline rumen temperature, it seemed logical that the differences between the physiological volume of the rumen at dissection and the actual volume of the contents in the rumen at the time of each drink contributed to the observed variation.

### Application of a correction factor to solve for an effective rumen volume

The results presented in Fig. 4 suggest that the *PV_r_*, measured in 26 sheep at slaughter, was smaller than the volume that a drink was mixed within the rumen. This discrepancy could have been due to rumen emptying during the time that the sheep were off feed prior to slaughter (Purser and Moir, 1966). It is also possible that the dynamics of heat exchange between the ingested water and the rumen contents violated the assumptions of the thermodynamic model, as we suppose occurred when heat was lost from the mixture during the bucket experiment. In the case of the animal experiments, heat was likely gained by the rumen/drink mixture from the body before the minimum rumen temperature occurred. Variation in the density and viscosity of rumen contents may also have influenced mixing efficiency, and ingested water may have been partially warmed during transit through the oral cavity and oesophagus prior to reaching the rumen. Together, these factors provide a plausible explanation for the divergence between theoretical predictions and the rumen temperature profiles observed in vivo. In the bucket experiment, heat loss from the mixture resulted in an overestimation of the volume of the drink by approximately 8%. In contrast, the animal house experiment underestimated the volume of the drink by approximately 50%. This discrepancy in the animal house experiments would have been due to heat gain by the rumen contents from the body, in contrast to heat loss from the bucket to the surroundings in the first experiment. Given that the temperature difference between the mixture and the surroundings in the bucket experiment was about 12 °C, and the average difference between the rumen and body in the animal house experiments was about 2 °C, the error induced by heat gain in the animal house experiment cannot account for the entire 50% error. More likely, the error in the animal house experiment was due to the measurement of rumen volume, which may not have adequately accounted for the density, viscosity, or thermal properties of the gut contents. These factors would influence the effective heat capacity of the rumen and, therefore, the magnitude and duration of the temperature change that was observed after a drink.

To circumvent these errors in estimation, we used the known volume of each drink, along with the measured values of minimum rumen temperature, baseline rumen temperature, and temperature of the drink, to calculate an “effective rumen volume” (*EV_r_*). The *EV_r_* is an estimate of the rumen volume that would have led to the observed change in rumen temperature when it was mixed with the drink. If the entire dataset was used to derive the *EV_r_* for each drink and we then used *EV_r_* to solve for the volume of each drink, a circular argument would have been created. Thus, the dataset from the animal house experiment was randomly divided into two parts. One-half of the data was used to solve for an *EV_r,_* and the other half to test whether the use of *EV_r_* improved the prediction of drink volume.

Using the first half of the dataset, the derived values for *EV_r_* were compared to the values for *PV_r_* to establish a correction factor to convert the *PV_r_* to *EV_r_*, and, thus, to correct Eq. 4 to predict *EV_r_* from *BM*. Equation 3 was then used with the derived *EV_r_* as the value for rumen volume to calculate the volume of each drink in the second half of the dataset and thus to assess the thermodynamic approach using *EV_r_*.

Across the first half of the dataset, *EV_r_* was consistently 1.92 times the value of *PV_r_*, indicating that the effective volume that a drink was mixed into in the rumen was substantially larger than the measured physiological volume of the rumen. Thus, we corrected Eq. 4 by 1.92 at the body mass of the smallest and largest sheep in the animal house experiment to derive Eq. 5, and thus estimate *EV_r_* from *BM*:


(Equation 5) $$EV_{r}\;(L)=(BM\times 0.24)-3.5$$

Equation 5 was then used to derive *EV_r_* for each sheep in the animal house experiment, and Eq. 3 was then solved to estimate the volume of water consumed at each drink.

**Figure 5.**
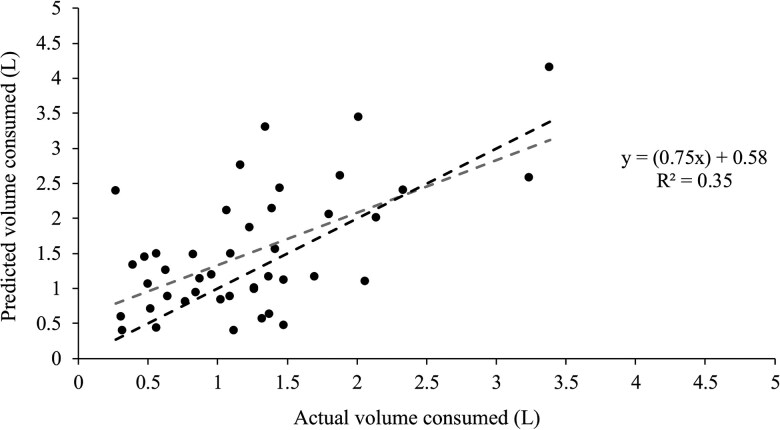
The actual volume of water consumed (L) during an individual drinking bout and the predicted volume of water consumed (L) using the effective rumen volume (***EV_r_*** derived using Eq. 5) in the thermodynamic equation (Fig. 1B; Eq. 3). The plot includes the line of identity (y = x;---) and the linear regression line (**---**).

Regression analysis of the estimated volume of water consumed at each drink against the measured volume of water consumed explained 35% of the variance (*r*^2^ = 0.35, Adj. *r*^2^ = 0.33, Mean Squared Error = 0.31, Root Mean Squared Error = 0.56 L, Mean Absolute Error = 0.45 L; Fig. 5). The relationship between the actual and predicted volumes was statistically significant (*P *< 0.01). On average, Eq. 5 overestimated a drink by 0.28 L. The *r*^2^ was lower than the model using *PV_r_* (Fig. 4; *r*^2^ 0.58), as might be expected for an analysis with only half the number of samples. However, the corrected model yielded a lower Mean Squared Error and mean bias, and the regression line was close to the line of identity (Fig. 5), indicating that the prediction was more accurate at an individual level, providing a more reliable estimate of water consumption even if the model did not fully explain the variance.

The predicted water intake was based solely on changes in rumen temperature. A proportion of ingested water bypasses the rumen and enters the abomasum, with estimates ranging from 18-60% in cattle and 10-30% in sheep (Woodford et al., 1984b; Cafe and Poppi, 1994). Given that the contents of the abomasum will exchange heat directly with the contents of the rumen across the abomasum wall, water that directly enters the abomasum should contribute to the observed change in temperature in the rumen, but the situation could contribute to the unexplained variance in the model. Fasting would be expected to accentuate this variance, as a reduction in the volume of digesta would alter both the rumen thermal capacity and the rate of fluid passage (Brod et al., 1982; Woodford et al., 1984a). In accordance with thermodynamic principles, any contribution from water that entered the abomasum would likely be expressed with a temporal delay relative to the direct cooling effect of water that entered the rumen.

### Water temperature, water consumption, and rumen temperature

**Figure 6.**
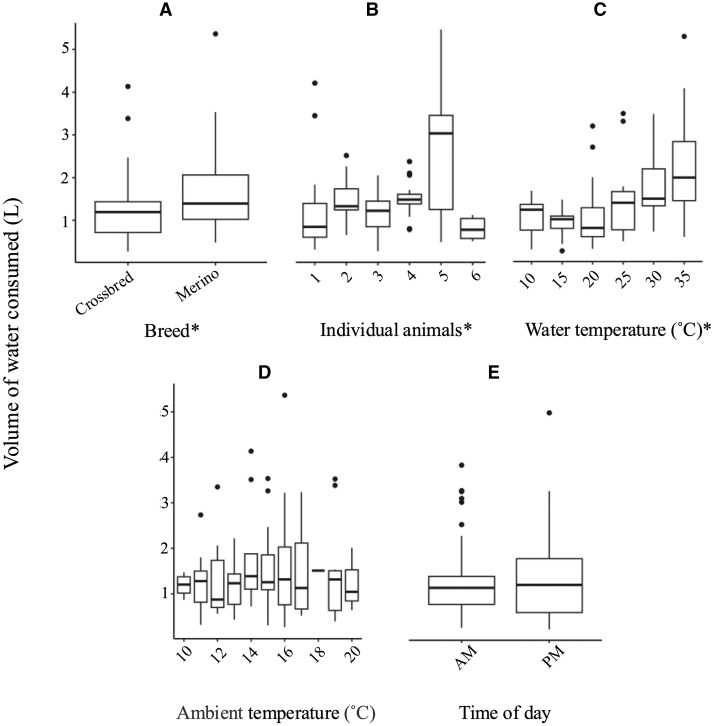
The effect of various parameters on water intake. Water intake (A) according to the breed of the individuals, (B) the six individual sheep (Crossbred; 1, 2 and 3, and Merino; 4, 5 and 6), (C) as a function of the water temperature, (D) as a function of the ambient temperature, and (E) as a function of time of day. Asterisks (*) on the x-axis indicate variables with a statistically significant effect on water intake (*P *< 0.05).

Breed had a significant effect on the volume of water consumed (*V_w_*) (Fig. 6A; *P *< 0.05), with Merinos drinking more water on average than the Crossbreds (1.73 L vs 1.24 L) during drinking bouts. The *V_w_* significantly differed between the individual animals (Fig. 6B; *P *< 0.01), possibly explained by the difference in individual metabolic rates based on breed, and on body mass, both of which influence water intake (Goldansaz et al., 2017; Barros de Freitas et al., 2021). Future studies could investigate how these individual differences can be measured and incorporated into predictive models to improve the accuracy of estimation of water intake, possibly using a combination of biometric data and metabolic profiling (Goldansaz et al., 2017).

Water temperature significantly impacted *V_w_*, with the largest volume consumed at 35 °C and the smallest volume at 10 °C (Fig. 6C; *P *< 0.01; 2.65 L vs 0.97 L). Ambient temperature did not significantly affect water consumption (Fig. 6D; *P *> 0.05). The study was conducted during winter, and the maximum temperature in the animal house during the experimental period was 20.5 °C. Therefore, it is likely that the sheep preferred warmer water to aid in thermoregulation while they were exposed to cool conditions (Al-Ramamneh, 2023). If this experiment was replicated in a paddock setting in warmer conditions, it is reasonable to assume that ambient temperature would have had a larger impact on *V_w,_* particularly if the ambient temperature was higher than 30 °C, with sheep consuming more water to maintain hydration when water is used for thermoregulation (Masters et al., 2023). The timing and volume of these drinking events would also be expected to shift under hotter conditions, which would influence the observed temperature dynamics and the accuracy of model predictions. Similarly, the composition and moisture content of the diet will also likely influence total water intake, with drier or more fibrous feed typically associated with higher water consumption (Araújo et al., 2010; Ammer et al., 2018; Barros de Freitas et al., 2021). The physical properties of the diet could also influence the heat transfer dynamics within the rumen, with denser or drier digesta potentially slowing the equilibrium of ingested water with rumen temperature. Time of day (AM or PM) did not impact water consumption (Fig. 6E; *P *> 0.05).

In commercial grazing systems, sheep consume a variable diet that is influenced by pasture availability, forage quality, and grazing patterns (Edwards et al., 1996; Shaw et al., 2006). The variability in feed will likely introduce more variability to the volume of the rumen, which will affect the robustness of any application of Eq. 3 (Fig. 1B). The proportion of liquid and solid content in the rumen will also affect the heat exchange properties in the rumen, influencing the rate at which water consumption changes the rumen temperature. A rumen with higher liquid content will have faster thermal exchange, whereas more solid contents will result in slower changes (Vesterdorf et al., 2022). Therefore, future experiments could aim to quantify the physical capacity of the rumen and its variability across breeds and body masses under commercial grazing conditions. Purser and Moir (1966) calculated the *PV_r_* using an approach similar to the approach in this study, however, they also measured the physical capacity of the rumen by completely emptying the contents of the rumen and reticulum and subsequently filling the rumen with water until it was fully distended, thereby determining the volume required to reach its maximum expansion. This method provided an estimate of the rumen’s total physical capacity, offering insight into the potential range of volumetric variability beyond physiological conditions. Understanding the interaction between diet composition and water intake patterns in commercial environments could provide critical insights to adapt the algorithm to extensive farming practices (Masters et al., 2023).

A limitation of this study was the small number of animals in the drinking experiments (n = 6), which reflected the labour-intensive nature of the design. With two breeds represented in this small cohort, the predictive power of the models was constrained. In addition, the rumen volume equation was derived from a relatively small sample size (n = 26), which is modest given the inherent variability in rumen capacity between animals. Future studies with larger and more diverse cohorts will be important to test robustness and improve generalisability.

**Figure 7.**
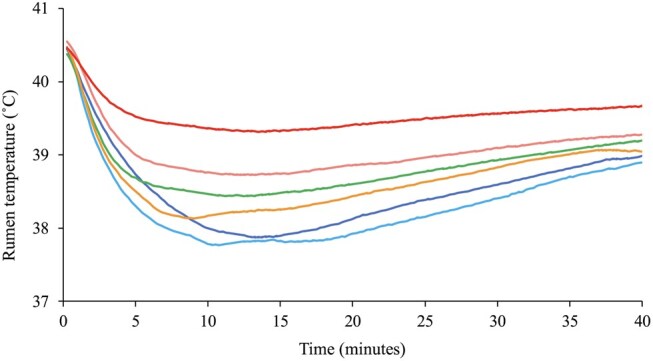
The mean change in rumen temperature of six sheep for 40 minutes after drinking water at each water temperature (10 °C [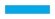], 15 °C [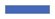], 20 °C [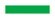], 25 °C [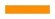], 30 °C [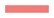] and 35 °C [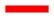]).

The mean baseline temperature of the rumen across the study was 40.5 °C, with the minimum temperature reached following a drink event ranging from 34.4 °C to 40.2 °C. On average, the decrease in rumen temperature was greater when water was at 10 °C was consumed compared to at 35 °C (Fig. 7; *P *< 0.01). A larger volume of water consumed resulted in a greater decrease in rumen temperature regardless of the water temperature (*P *< 0.01). The effect of water temperature and volume on the decrease in rumen temperature aligned with findings from Cantor et al. (2018), with larger volumes of colder water causing greater temperature changes, and smaller, warmer drinks having less effects. These findings have practical implications for on-farm water management for ruminants, suggesting that providing cooler water in larger volumes could influence rumen temperature and support thermoregulation, particularly during periods of heat stress.

Precision agriculture is revolutionising livestock management by integrating data sources and technologies to improve animal welfare and streamline the remote monitoring of livestock (Schillings et al., 2021). Through remote sensors, GPS tracking, accelerometers, and environmental data, farmers can gather real-time information on animal health, behaviour, hydration levels, and environmental conditions, providing a holistic view of animal health (Masters et al., 2023). This data-driven approach allows for more informed decision-making to help farmers optimise the management of water and feed, to monitor heat stress, and to detect early signs of disease (Schillings et al., 2021). Similarly, research into the physiological adaptations of livestock to extreme climates is crucial in the face of climate change. Understanding how animals cope with heat stress, dehydration, and other climate-related challenges is vital to develop strategies to mitigate these impacts (Masters et al., 2023).

Body mass, or liveweight, is a readily accessible metric in Australian sheep farming. In an extensive farming system, if rumen temperature is monitored, then the application of Eq. 5 will allow the prediction of *EV_r_* without the need for rumen dissection, and then Eq. 3 can be used to derive the volume of a drink from the change in rumen temperature caused by the drink, and, therefore, the volume of water consumed by sheep. This information will be vital in understanding how sheep adapt physiologically to heat stress, particularly during extreme heat events when ambient temperatures exceed 30 °C (Hopkins et al., 1978; Kumar et al., 2017).

## Conclusion

To our knowledge, this is the first study that has investigated the impact of heat transfer during water consumption on changes in rumen temperature to estimate the volume of water consumed. Our findings demonstrate that a simple thermodynamic equation can be adapted to estimate water intake from temperature changes recorded by an indwelling rumen logger. However, limitations in the algorithm’s robustness, particularly related to inaccuracies related to the rumen volume, must be addressed. Future iterations of the algorithm could incorporate corrective factors to account for variability in rumen fluid viscosity, logger positioning, environmental conditions, breed, and the water content of the feed. Validation in paddock environments, where drinking episodes cannot be directly observed, will ensure the algorithm’s practical application. Once refined, this algorithm has the potential to serve as a valuable tool to monitor water intake and symptoms of heat stress in sheep, guiding management decisions to optimise productivity and welfare in the Australian sheep industry.

## Abbreviations:

*BM*: body mass of the sheep
*T*_1_: temperature of the simulated drink (Bucket 1)
*T*_2_: temperature of the simulated rumen (Bucket 2)
*T*_1 + 2_: temperature of the resulting mixture (Bucket 1 + Bucket 2)
*V*_1_: volume of the simulated drink (Bucket 1)
*V*_2_: volume of the simulated rumen (Bucket 2)
*PV*_1_: predicted volume of the simulated drink (Bucket 1)
*V*_r_: physiological rumen volume
*PV*_r_: predicted physiological rumen volume
*EV*_r_: effective rumen volume
*PV*_w_: predicted volume of water consumed
*T*_r__-base_: baseline rumen temperature before a drink
*T*_*m*_: minimum rumen temperature after a drink
*T*_w_: temperature of the water consumed
*V*_w_: volume of water consumed
CCTV: closed-circuit television
